# Diffusing a Research-based Physical Activity Promotion Program for Seniors Into Diverse Communities: CHAMPS III

**Published:** 2006-03-15

**Authors:** Anita L Stewart, Dawn Gillis, Melanie Grossman, Martha Castrillo, Barbara McLellan, Nina Sperber, Leslie Pruitt

**Affiliations:** Institute for Health & Aging, University of California, San Francisco; Institute for Health & Aging, University of California, San Francisco, San Francisco, Calif; Institute for Health & Aging, University of California, San Francisco, San Francisco, Calif; Institute for Health & Aging, University of California, San Francisco, San Francisco, Calif; Institute for Health & Aging, University of California, San Francisco, San Francisco, Calif; Institute for Health & Aging, University of California, San Francisco, San Francisco, Calif; Institute for Health & Aging, University of California, San Francisco, San Francisco, Calif, Stanford Prevention Research Center, Stanford University School of Medicine, Stanford, Calif

## Abstract

**Introduction:**

Increasing the physical activity levels of older adults through diffusion of successful research-based programs into community settings is challenging because of differences between research and real-world settings. This project diffused the Community Healthy Activities Model Program for Seniors (CHAMPS) II, an individual-level research-based physical activity promotion program, through three community organizations to reach lower-income and minority (primarily Hispanic or Latino and African American) seniors.

**Methods:**

Through an academic–community partnership, university staff worked with each organization to adapt the program to be appealing and effective, enable their staff and volunteers to provide the program, increase participants' physical activity, and leave sustainable programs in place. Evaluation was based on methods recommended by the Centers for Disease Control and Prevention.

**Results:**

The adapted programs, referred to as CHAMPS III, differed from the original program and among organizations. Group-based components and resource guides were included and new features were added; however, individualized components were not offered because of limited resources. A total of 321 people enrolled among three organizations; there was a trend toward increased physical activity at two organizations (an estimated increase of 481 kcal/week [*P* = .08] and 437 kcal/week [*P* = .06] expended in physical activity). Evaluation revealed challenges and unexpected community-level benefits. All organizations are continuing efforts to promote physical activity for older adults.

**Conclusion:**

This project enabled community organizations to implement physical activity promotion programs. The overarching challenge was to retain original program features within each organization's resources yet be sustainable. Although the programs differed from the original research program, they were a catalyst for numerous community-level changes. Our findings can guide similar projects to reach underserved older adults.

## Introduction

To address the current public health problem of widespread sedentary behavior among older adults in the United States, numerous initiatives have emerged to promote increased physical activity (PA) (1,2). These are based on substantial evidence of physical and mental health benefits of regular PA for older adults (1,3,4), including walking (5,6).

Evidence for the effectiveness of community-level interventions to increase adult PA was reviewed by the Task Force on Community Preventive Services (7). Two "strong" recommendations were individually adapted health behavior-change programs and creating or improving access to places for PA, particularly in neighborhoods with the least resources, combined with informational outreach. Individually tailored PA programs and interventions that include principles of behavior change were also featured in the recent Best Practices Statement for promoting PA in older adults developed by a coalition of national organizations led by The American College of Sports Medicine (8).

Many initiatives emphasize the need to increase PA in underserved populations, such as racial and ethnic minorities or people with low socioeconomic status (SES) (1,9), primarily because these groups are at higher risk of poor health (10-14) and have lower levels of PA than their counterparts (15,16). For example, among adults aged 65 and older who participated in the National Health Interview Survey, 2001–2002, only 13% of non-Hispanic blacks and 13.6% of Hispanics or Latinos reported regular leisure-time PA, compared with 22.8% of non-Hispanic whites (17).

One promising approach to increasing PA among older people in underserved populations is to diffuse into the community successful research-based PA-promotion programs that are consistent with the recommendations of the Task Force on Community Preventive Services and the Best Practices Statement (8). However, there are few methods or guidelines for adapting and diffusing such programs (18). In addition, results from ethnically inclusive studies are often not disseminated widely (19). Closing statements from The Cooper Institute conference in 2004, "Increasing Physical Activity in World Populations: Understanding Diffusion and Dissemination," suggested that diffusion and dissemination are areas that are still developing (20). 

This article reports on a project to diffuse a research-based PA-promotion program, the Community Healthy Activities Model Program for Seniors (CHAMPS) (21), into three community organizations to reach primarily lower-income, racial and ethnic minority older adults. CHAMPS successfully increased PA in adults aged 65 and older in two research studies funded by the National Institute on Aging: CHAMPS I was conducted in congregate housing settings (22), and CHAMPS II was a randomized controlled trial of sedentary or underactive members of Medicare health maintenance organizations (23).

The goal of CHAMPS III was to evaluate the efficacy of diffusing CHAMPS II by implementing it in three community organizations, adapted to their context and clientele. Specific objectives were the following: 1) to work with staff from participating organizations and community members to adapt CHAMPS II to be appealing, feasible, and sustainable; 2) to diffuse the modified programs by enabling their staff and volunteers to conduct the programs, with training and assistance; 3) to evaluate implementation processes; 4) to increase participants' PA levels; and 5) to leave programs in place at each organization. We describe these processes and challenges as well as ideas for improving translation and diffusion processes in other settings. Our results contribute to a small body of literature on diffusing PA-promotion programs to reach underserved populations.

## Methods

### Research basis

CHAMPS is a lifestyle PA-promotion program for older adults. The choice-based individually tailored program provides information, skills training, support, and problem solving through a personal planning session, regular telephone follow-up, group workshops, newsletters, activity diaries, and functional fitness assessments. Using principles of social-cognitive theory, trained staff members assist participants to develop and maintain a PA regimen of their choice, based on their health, preferences, readiness to increase activity, ability, and resources. Participants are encouraged to join existing community-based PA classes and programs, develop a regimen on their own (with guidance), or both. They learn how to motivate themselves, overcome barriers, exercise safely, develop a balanced program (endurance, strength training, flexibility, balance, and coordination), and progress slowly.

### Community–campus partnerships

For CHAMPS III, the University of California, San Francisco (UCSF) partnered with three San Francisco Bay Area community organizations differing in purpose, size, infrastructure, and clientele. The organizations were as follows:

- Network for Elders ("Network") in Bayview Hunters Point, San Francisco, provides case management and referral for about 500 frail elders and their families to help elders remain in their homes and reduce isolation. An affiliated interfaith volunteer program provides many services. The city of San Francisco has designated the region as a low-income concentration area and an area of concentration for African Americans and Asian/Pacific Islanders.
- On Lok's 30th Street Senior Center ("30th Street") in the Mission District of San Francisco provides approximately 250 seniors per day with a range of activities and services to maintain or improve their well-being. Volunteers offer many of the services. The city of San Francisco has designated the region as a low-income concentration area and an area of concentration for Latinos. 
- Sequoia Hospital Health & Wellness Services, a hospital-affiliated outpatient center in Redwood City, serves parts of San Mateo County. It provides wellness programs to promote the independence and well-being of adults, in partnership with physicians. Although its clientele tends to be nonminority (its service area was 38% nonwhite in 2003), it aimed to recruit lower-income, minority older adults for this project.  

The organizations were selected to represent different organizational models for program diffusion to reach underserved populations. Selection was also based on a history of collaboration with UCSF faculty working in minority health research. Because extensive time is needed to establish an effective relationship with community-based organizations (24,25), this history helped to provide a foundation of trust. We aimed for each organization to provide the program to community members, with UCSF serving in a background role. 

The UCSF team had expertise in social work, exercise physiology, health education, and community work. UCSF's role was to 1) help adapt the program to be as similar as possible to the original, 2) develop new program materials, 3) translate materials into Spanish as needed, 4) provide training, 5) monitor implementation, and 6) conduct program evaluation.

### Program evaluation framework

The California Endowment funded the project in November 1999 through its CommunitiesFirst initiative (26). This initiative was not intended to support research, so the goal of CHAMPS III was to develop sustainable programs that met the needs of the communities and organizations. The evaluation was designed to reflect the entire process — from adaptation through implementation. The desire of our three organizations to minimize the burden of assessment (i.e., paperwork) for participants and staff guided many of our choices. We reviewed several program evaluation approaches (27) and selected the Centers for Disease Control and Prevention's *Physical Activity Evaluation Handbook* (28), which conceptualizes a project as a sequence of steps leading to a specified goal. A logic model describes influential factors, inputs, activities, and initial, intermediate, and long-term outcomes, including links among them. Each step is evaluated, and a feedback loop is included. The logic model is intended as a flexible framework that can be interpreted to fit specific project needs. The Figure presents our interpretation of the logic model for CHAMPS III.

### Inputs and planning activities 

The project included 1 year for planning and adaptation (November 1999 through October 2000) and 2 years for implementation and evaluation (November 2000 through October 2002). Planning and adaptation activities included community focus groups on PA attitudes, preferences, and barriers (29,30); planning meetings; and advisory groups (Figure).

**Figure 1:**
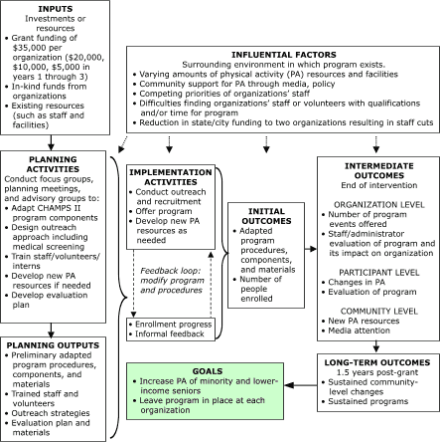
Logic model for evaluating diffusion of the Community Healthy Activities Model Program for Seniors (CHAMPS) II to reach racial and ethnic minority and lower-income seniors (CHAMPS III).

**Table:** 

### Planning outputs 

We adapted the program for each organization to maximize safety, feasibility, sustainability, and effectiveness. The original model was not feasible for any of the three organizations selected for this study; thus, during adaptation, we identified the purpose of each component of the original program (e.g., education, motivation) and determined whether it could be achieved in a different way. For example, although grant funding was available, 30th Street did not hire an exercise specialist because it was not sustainable. (Volunteers provide many of their programs.) Adaptations continued during implementation based on participant and staff feedback. Planning outputs for each organization are summarized in Table 1.

One challenge was determining appropriate medical screening procedures based on staffing differences and a reduced capacity for providing individual guidance compared with CHAMPS II. Unique situations also had to be considered; for example, most PA classes at 30th Street were historically available without medical screening or consent. Balancing the need for participant safety with the need to reduce barriers to participation also influenced CHAMPS III procedures. 

The evaluation plan was similar among organizations and included process evaluation to understand adaptation and implementation (31). Every 6 to 12 months, the principal investigator conducted informal, semistructured interviews with staff, volunteers, and directors to obtain their perspective on the successes and challenges of the program and suggestions for improvement. Depending on the project phase, we included additional questions on topics such as collaboration, sustainability, and the program's effect on the organization and community at large.

To measure individual outcomes, interviewers administered the CHAMPS Physical Activity Questionnaire (21,32-34) in English or Spanish at enrollment and 6 months. From these data, we estimated for each cohort the mean number of calories per week expended and hours per week spent in PA. We compared baseline and 6-month data using paired *t* tests to determine the significance of the changes. Staff also obtained informal input from participants throughout the project, and the Network director conducted a discussion group at the end of the first cohort. The UCSF Institutional Review Board approved a waiver of consent for all evaluation procedures.

## Results

We report on the implementation of CHAMPS III as well as initial, intermediate, and long-term outcomes. Initial outcomes include differences between CHAMPS II and the programs offered by each of the three organizations in CHAMPS III and the sociodemographic characteristics of the participants at each organization. Intermediate outcomes were measured at the end of the intervention and included data on participant levels of PA and organization- and community-level changes. Long-term outcomes were measured 1.5 years after funding for CHAMPS III ended.

### Implementation activities

Each organization offered a 6-month program, enrolling two to seven cohorts. During implementation, Network and 30th Street required substantial assistance from UCSF staff, whereas Sequoia conducted its entire program using its own resources. Because PA resources were almost nonexistent in Network's community, developing new resources became an essential part of its program.

Feedback from participants and staff and other factors such as community interest resulted in some early recruitment and program modifications. For example, at Network and 30th Street, the initial recruitment plan was adapted to provide "rolling" or ongoing enrollment to accommodate individuals who wanted to join after the program was underway. 

### Initial outcomes

Table 2 shows how each program in CHAMPS III differed from CHAMPS II in several areas, including staffing, duration, and components offered, and the manner in which the components were provided. The lack of an exercise specialist and other health professionals to offer the one-on-one program components at 30th Street and Network represented a major change from CHAMPS II to CHAMPS III, and the program was shortened from 1 year to 6 months. Of the components offered in CHAMPS II, the following three were included at all CHAMPS III organizations: functional fitness testing, group workshops, and PA resource directories. Functional fitness assessments were conducted at two sites by volunteers or other participants for educational, planning, and motivational purposes only.

Only Network provided newsletters but in a modified form. 30th Street created a program bulletin board to display announcements and information. The personal planning session and ongoing telephone support were not offered by any organization because of staffing and budget constraints. In CHAMPS II, these two components enabled personal attention and individualized guidance through discussion of topics such as readiness to change, exercise safety, barriers to PA, resources, and goal setting. Even Sequoia found these components too labor-intensive despite having appropriate staff. Elements from these components were included in CHAMPS III group workshops whenever possible. All organizations used occasional reminder telephone calls; Network also provided check-in calls when participants missed activities. New features were added to some programs, such as a weekly program-based exercise class at Network. This class provided informal support activities, such as sending get-well cards to fellow participants.

Table 3 summarizes the characteristics of participants at the three organizations, which enrolled a total of 321 people; most (76% to 97%) were women. A broad age range was represented: 24% to 52% were aged 75 years and older. Each organization had a different ethnic mix: of Network's enrollees, 90% were African American; of 30th Street's enrollees, 84% were Latino. Sequoia had difficulty recruiting lower-income and minority groups; however, of its enrollees, 33% were nonwhite. Participants at 30th Street had low educational levels; more than half had less than a high school education. At Sequoia, just over 10% had less than a high school education; education was not assessed at Network because community input indicated that the question was too sensitive. Each organization promoted some events to the community at large (or the entire senior center); therefore, the reach of each program sometimes extended beyond the people who formally joined.

### Intermediate outcomes 

Intermediate outcomes were measured at the organizational, participant, and community levels immediately after the intervention was completed.

**Organization-level outcomes**

Each program was unique, and offerings changed as the program evolved and new cohorts were enrolled; thus, the number of activities that were offered varied. Network's cohorts could attend seven or eight exercise- and health-related workshops and a weekly exercise class. Functional fitness tests were offered two times per cohort. Special events like potluck luncheons were held occasionally. 30th Street offered to each cohort six exercise-related workshops, two to three additional exercise- and health-related presentations, five to seven *The Doctor Is In* sessions (a question-and-answer forum with a physician about exercise and various medical conditions), and one to four special events such as celebrations of participants' program completion. Participants could complete fitness testing one or two times. Sequoia's program at each senior center consisted of eight workshops, including two functional fitness testing sessions and a celebration at completion of the program.

We obtained input on intermediate organization-level outcomes from staff and administrators through process evaluation. A Network administrator noted that although the organization primarily focused on frail elders, CHAMPS III provided an opportunity to reach seniors who were not homebound or as frail as its typical client. The project also brought together public and nonprofit community organizations.

30th Street administrators reported positive organization-level changes such as enhanced awareness among staff, volunteers, and seniors of the benefits of PA as well as increased participation in their ongoing exercise classes (open to all 30th Street attendees). The emphasis on increasing PA also attracted younger seniors to the center. Some local physicians referred patients to 30th Street because of increased program awareness. Because of low participation among men, 30th Street hosted a men's conference on the benefits of PA and sought information on what might help them become more active. Efforts were then initiated through the city's recreation and parks department to develop activities suggested by the men. New partnerships provided a channel for sharing information on topics such as potential funding or community resources. Both 30th Street and Network gained access to several academic programs that provided student interns.

Sequoia's new approach of offering programs at community senior centers (rather than at its own site) led to increased community awareness and appreciation. Sequoia created new partnerships and strengthened existing ones with several senior centers; they are committed to working together to help maintain seniors' independence.

**Participant-level outcomes**

Of the 321 participants enrolled in the three programs, 207 completed baseline and 6-month follow-up questionnaires. Six-month changes in PA are shown in Table 4, indicated by changes in estimated caloric expenditure per week and hours spent per week in PA. A trend toward increased PA was observed at Network (+481 kcal/week, *P* = .08; +1.9 hrs/week, *P* = .10) and Sequoia (+437 kcal/week, *P* = .06; +2.0 hrs/week, *P* = .08). No statistically significant change in caloric expenditure was observed at 30th Street, but there was a trend toward decreased duration (−1.4 hrs/week, *P* = .09). For the total sample among organizations (n = 207), there was a trend toward increased caloric expenditure (+213 kcal/week, *P* = .10). The overall 6-month response rate at Sequoia was low (52%), with 83 of 160 participants completing the follow-up questionnaire; thus, results may not represent all people participating in the Sequoia program. The response rate at Network was 85% (53/62), and the response rate at 30th Street was 72% (71/99).

Network obtained an informal evaluation by participants through a one-time discussion group after being in the program for about 6 months. Most of the feedback was positive. For example, the participants particularly liked the group exercise, fellowship, and a chance to meet new people. They felt that the program "helped them help themselves" and perceived both physical and mental benefits. Constructive criticism was also provided; participants felt that the classes and workshops were too short, and they wanted more exercise equipment such as treadmills. 

**Community-level outcomes**

At Network, new partnerships with other community organizations developed, enhancing PA resources for seniors. As noted previously, there were no exercise classes available in the Bayview Hunters Point community at the beginning of the program. By the end, there were six: three offered by the local community college, one beginner pool-exercise class, and one African American line-dancing class, in addition to the program's exercise class. One intern helped start a walking group that developed into a partnership between the program, the city's recreation and parks department, and Network's interfaith volunteer group. All of these exercise resources were formally or informally associated with Network's program. Program participants regularly attended the community college classes and helped keep enrollment high enough to sustain the classes. Seniors and staff in Network's program successfully advocated for new chairs, improved lighting, and regular cleaning of the gymnasium where some classes were held. Some enrollees became formally involved with the Trust for Public Land in developing a new community park. The Trust approached the seniors to conduct programs in the park, plan clean-up and planting days, and promote these activities in their community.

At 30th Street, a new tai chi class was offered twice weekly and became affiliated with the program, with an average attendance of nearly 40. During the project, Sequoia's staff learned from participants that there was a need for fall prevention; some participants dropped out because they were afraid of falling. Sequoia developed and started teaching fall-prevention and strength-training classes at three senior centers. The new program attracted new clients. One senior center organized a fitness advisory board to determine which fitness and health classes should be offered.

Each program received news media attention that helped expand its reach into the community, although most attention was for Network. A local community newspaper published four articles about PA and Network's program, and the program was featured on KQED (National Public Radio) in a prime-time report. The line-dancing class was featured by the *San Francisco Chronicle*. For 30th Street, a local Spanish-language newspaper included an article about PA for seniors and the 30th Street program.

### Long-term outcomes 

Long-term outcomes are defined as those that were measured 1.5 years after the funding for CHAMPS III ended. Ultimately, the success of diffusion programs is determined by their sustained availability to community members; all organizations in CHAMPS III are continuing efforts to promote PA for older adults, despite funding challenges.

At Network, five community dance and exercise classes have continued. In addition, Senior Strutters, a performance group that evolved from the African American line-dancing class, often performs at community events. Because of the increased number of seniors at the city recreation and parks department's facility where the program class is held, the department provided a staff member to help with set-up and registration, and an employee now teaches the class, which may help the class continue. Network staff maintains the monthly PA resource calendar discussed previously. These are substantial accomplishments, because the entire program is run by the seniors who volunteer their time to promote and coordinate the program and sustain the exercise classes created in their community; no staff is currently paid to coordinate the program.

30th Street obtained funding for 2 years to continue and expand its program. The grant supported a bilingual Wellness Services Coordinator dedicated to the program and provided for two treadmills. In the new program, physician consent was obtained using a new form that enables physicians to indicate which classes offered at 30th Street are appropriate for each individual. This allows the coordinator, without being a trained exercise professional, to tailor the program to some extent for each participant. In addition, 30th Street's tai chi class has continued, and a second advanced class has been added.

At Sequoia, efforts have shifted to providing the *Mature & Secure From Falls* program. Sequoia Hospital offers these programs at no charge. The program has developed into a 6-week community-based class series and has been presented at more than 20 sites in San Mateo County. Sequoia also convened the San Mateo County Fall Prevention Task Force, where 27 organizations providing services to seniors are working to increase awareness about the need for fall prevention and to develop related resources.

## Discussion

CHAMPS III provided insight into the process of translating and diffusing a research-based program into diverse community-based organizations to reach underserved older adults. We are aware of only a few studies that have diffused research-based PA programs to reach minority and lower SES subgroups of older adults. The California Active Aging Program was a large-scale effort to reach seniors through a variety of community settings; although not focused on underserved people, it reached a diverse sample (36). Active for Life is a program to establish research-based PA programs in community settings for adults aged 50 and older; 2 of the 10 settings focus on minority and lower SES individuals (37). Our study differs from the California Active Aging Program in that, together with our community partners, we obtained community input in our planning year and throughout the project (as part of the participatory research model) to develop adapted programs. Further, we provide more extensive process evaluation information, including challenges and solutions related to diffusion. Last, we documented unintended community-level changes that occurred in response to the program. 

Our original intent was to diffuse CHAMPS II with some adaptations to meet the needs of the communities and organizations; however, it became apparent that the program would need to change substantially to be feasible and sustainable. The most substantial changes were the loss of the personal planning session and ongoing motivational telephone support at all three organizations and the lack of a staff exercise specialist at two organizations. Without these components, the programs were not able to provide the ongoing, individual-level guidance and support available in CHAMPS II.

We speculated on why we did not observe more changes in PA levels. It is difficult to compare preintervention and postintervention PA changes between CHAMPS III and CHAMPS II because CHAMPS II was a randomized controlled trial. Nevertheless, in CHAMPS II, the 1-year estimated increase of 687 kcal/week in PA among the intervention group (23) was much greater than the estimated increase of 213 kcal/week achieved in the total CHAMPS III sample. Program adaptations likely contributed to these differences; whereas CHAMPS II offered most components recommended in the Best Practices Statement (8), many of these were lost with the adaptations. Other possible explanations are that CHAMPS II excluded people who were already physically active, whereas physically active people were able to join CHAMPS III. The baseline level of energy expended in PA among CHAMPS III participants was 2545 kcal/week; among CHAMPS II participants, the baseline level was 1052 kcal/week (23). The California Active Aging Program, using a pre–post design, demonstrated total estimated increases of 644 kcal/week (midpoint) and 707 kcal/week (endpoint) (36). However, that program included individuals aged 50 and older, included more program components than CHAMPS III, and also excluded people who were already active.

Also of interest is why Network and Sequoia had trends toward increased levels of PA, but no change or a trend toward decreased PA (depending on the measure) was observed at 30th Street. Many seniors at 30th Street may have joined because they happened to be at the center at the time of enrollment and fitness testing, and they wanted to be part of what was happening that day. At Network, individuals had to go out of their way to join and participate. Some participants at 30th Street did not feel comfortable joining existing classes because they were concerned about exercising with certain medical conditions or limitations; they requested a weekly program class in which they could get guidance similar to that given in the monthly workshops by the exercise physiologist. Network's class was taught by an exercise physiologist and provided social support. Scheduling conflicts related to the participant's transportation to the center, volunteer duties, extended travel to their country of origin, and difficulties with keeping track of when program events were held (because they weren't on a weekly schedule) seemed common at 30th Street and may have contributed to the PA findings.

Sequoia's variability in PA changes among cohorts is more difficult to interpret. The variability highlights the unique challenges of collecting evaluation data for a program offered by community organizations (rather than universities such as UCSF). Because program funding was not focused on research, we had to limit paperwork, including questionnaires. Sequoia's lower response rates may have been partially responsible for the variability. Sequoia chose to collect data without assistance from UCSF, making quality control difficult. In fact, Sequoia's staff reported that collecting data on PA was one of the main challenges of the project. At the other sites, extensive efforts were required to collect data; UCSF staff worked closely with organization staff to find participants and administer the questionnaires face-to-face. Overall, it was difficult to provide a practical community-based program while obtaining useful evaluation data. At times, the amount of missing or questionable data precluded our reporting the findings (e.g., data related to participation in program events). Future programs should address these issues in the grant-proposal stages. It would help to designate and fund university staff to collect all data.

Ideally, more funding should also be allocated toward a more extensive development phase for diffusion project proposals. Although we met with organization administrators before submitting our grant, it was only after the grant was funded and numerous planning meetings took place that both sides truly understood the other's expectations and resources. It then became apparent that UCSF staff would need to play more substantial roles than planned at two sites. This shift raised sustainability concerns, but it was important to support the organizations in offering some version of CHAMPS and observe how it could be sustained. We also learned that some organizations would have preferred to be offered an existing program rather than developing it together; that is, they wanted to get a program going quickly and respond to problems when they emerged. Because much literature emphasizes the need to involve the community in all aspects of planning (38,39), we were surprised by this feedback.

Based on our experiences, this project succeeded because of at least one enthusiastic staff member at each organization committed to helping seniors with this program. In addition, UCSF staff characteristics that helped were being 1) culturally competent, 2) interested in being involved in the community beyond project demands, 3) creative and patient in overcoming barriers, and 4) able to adapt on a moment's notice. For future projects, it would be helpful to have university or organization staff members serve as a liaison to make connections among resources. In a related study, we found that many community organizations are interested and willing to help in many capacities (e.g., developing new PA resources); however, someone needs to cultivate relationships and translate intentions into action (40).

We consider the community-level changes effected through implementation of an individual-level program to be the most successful part of our project. This project enabled three organizations to adapt and implement a program to increase PA among minority and lower-income seniors. The three organizations are committed to retaining PA as part of their offerings; Network and 30th Street have sustained some program components, and Sequoia is promoting PA through its new fall-prevention program.

The main caveat is that the project was only a first step toward a sustained effort. To adequately diffuse such a program into communities to serve lower income and minority older adults, settings are needed with more infrastructure and capacity, with staff experienced in reaching and working with culturally diverse groups, and with an exercise specialist available to offer parts of the program. An alternative model is to partner with public health departments offering chronic disease prevention programs, where health educators and public health nurses might provide program features with assistance and input from exercise specialists (40). Public health departments already serve precisely the seniors we targeted; thus, they already have established relationships in the community and mechanisms for conducting outreach.

This article provides an example of applying the program evaluation approach from the *Physical Activity Evaluation Handbook* (28) to a diffusion project. We have highlighted the complexities of evaluating community programs to reach minority and lower-income older adults. Our findings should facilitate the diffusion of other programs such as CHAMPS into communities to reach some of our nation's more vulnerable older adults.

## Figures and Tables

**Table 1:** Planning Outputs for Community Organizations Participating in the Community Healthy Activities Model Program for Seniors (CHAMPS) III

**Planning Output**	**Network for Elders**	**On Lok's 30th Street Senior Center**	**Sequoia Hospital Health & Wellness Services**
**Program location**	City's recreation and parks department gymnasium	On site	On site initially, then at four senior centers in Sequoia's service area
**Trained staff and volunteers**	No one with the needed expertise was available for one-on-one program components. University of California, San Francisco (UCSF) staff conducted a series of nine 1.5-hour training sessions for volunteers to serve this role. Eight volunteers completed training but were better prepared for and preferred to help with transportation and recruitment. Student interns trained by UCSF staff assisted with program implementation. A licensed practical nurse worked 15 hours per week as program coordinator and assisted with implementation and reminder and check-in phone calls.	No one with the needed expertise was available for one-on-one program components. UCSF staff conducted a series of nine 1.5-hour training sessions for volunteers to serve this role (plus two follow-up sessions and three extra sessions for fitness testers). Twenty volunteers completed training but were better prepared for and preferred to help with recruitment, fitness testing, and reminder phone calls. A program and activities coordinator helped organize program events and recruit participants; an assistant helped advertise events and track activities.	Sequoia had staff members with the needed expertise but decided to eliminate one-on-one program components because they were too labor-intensive. An exercise physiologist coordinated and conducted the program with assistance from other staff (a nurse and dietitian). Staff and volunteers at the various senior center sites (where they conducted their program) helped with recruitment, promotion, and reminder telephone calls.
**Outreach strategies**	Recruited participants initially through volunteer membership; later presented information on physical activity and health at several community venues. Promoted also through word of mouth, articles in existing newsletter and local paper, and affiliated community exercise classes.	Organized special events to enhance awareness, including plays in which seniors dramatized the benefits of the program and how physical activity could improve their health. 30th Street and UCSF staff personally invited many individuals at the center to attend program events.	The senior centers (where programs were provided) recruited through flyers and word of mouth. The program was advertised in the Sequoia Hospital Community Calendar, local newspapers, and the city's recreation guide. A videotaped talk by a popular geriatrician on a local television channel provided program contact information.
**Medical screening procedures**	Initially required physician consent but progressed to a medical screening form and blood pressure measurements that allowed lower-risk participants to start the program while their physicians were notified of their enrollment. Staff reviewed safety tips and other items in newcomers' folders as they enrolled. Safety aspects covered in workshops were added to each program exercise class.	A medical screening determined whether physician consent was required for functional fitness testing, but physician consent was required to perform (versus observe) the moderate-intensity exercise portion of the workshops. Early workshops involved only light stretching; thus, participation was allowed while consent was obtained.	Medical screening questions and blood pressure screening determined if physician consent was required for functional fitness testing. However, all workshops were lecture based without participatory exercise, so physician consent was not required for the program. Sequoia used these procedures to reduce staff burden and avoid the potential barrier associated with requiring physician consent.

**Table 2:** Program Adaptations of the Community Healthy Activities Model Program for Seniors (CHAMPS) II at the Three Community Organizations Participating in CHAMPS III

**Original Program Features**	**CHAMPS II**	**CHAMPS IIIOrganization and Program Name**		

	**Palo Alto Medical Foundation**	**Network for Elders**Seniors in Motion for Health	**On Lok's 30th Street Senior Center**Always Active/*Siempre Activo*	**Sequoia Hospital Health & Wellness Services** Aging With Energy
**Duration**	1 year	6 months	6 months	6 months
**Functional fitness testing^a^**	University of California, San Francisco (UCSF) staff initially conducted testing before enrollment as baseline data and as part of medical screening; repeated at 6 months (midpoint) and 12 months (endpoint).	UCSF staff offered at baseline and 6 months for evaluation as well as for educational and motivational purposes.	Offered by trained volunteers (with supervision) for recruitment, educational, and motivational purposes at baseline and 6 months for the first cohort and only at baseline for the second cohort.	Sequoia staff helped participants test one another at baseline and 6 months. Results were used in workshop discussions about improving fitness and function and making an exercise plan and at 6 months to discuss changes.
**Group workshop series**	UCSF staff offered a series of 10 exercise- and health-related workshops, which provided tips to keep exercise safe and an opportunity for supervised practice of different types of exercise. Topics such as overcoming barriers were discussed in small groups.	UCSF staff offered a series of seven to eight exercise- and health-related workshops. We reduced content and provided a more interactive format. By the end of the second cohort, much of the workshop material was incorporated into the program exercise class. Additional health-related workshops were offered by guests.	UCSF staff offered a series of 6 exercise-related workshops. Additional health-related workshops were offered by staff or guests. We reduced content and provided a more interactive format to accommodate bilingual presentations and lower levels of education and literacy.	Sequoia staff offered their own series of 6 exercise- and health-related workshops at various community senior centers or sites involved with the project. These workshops included exercise demonstrations rather than participatory exercise.
**Community physical activity resource directory**	A directory of ongoing physical activity (PA) resources in the community was created by program staff and provided to participants. Counselors discussed the guide with seniors who expressed interest in classes and programs.	A directory of ongoingphysical activityactivities in the community, as well as television and Web offerings, was developed and distributed to participants; it was updated every 4–6 months.	A community directory was not available; participants received a monthly calendar of program activities, and each day the center posted a list of its classes and activities on a display board.	A community directory was not available; however, each senior center promoted its own classes and offerings. These were also mentioned in some workshops.
**Newsletters**	Monthly newsletters that highlighted participants' accomplishments, provided tips, announced workshops, and included other exercise- and health-related information were mailed to participants.	Although not program-specific, a monthly calendar of community physical activity opportunities and occasional exercise- or program-related articles were added to an existing community newsletter, distributed to about 300 individuals or organizations.	No program-specific newsletter was produced, but information about program activities was posted on a new *Always Active* bulletin board at the center.	Not offered
**Activity diaries or logs**	Participants completed 2 weeks per month, mailed to counselors, and discussed during telephone support.	Not offered	Not offered	Not offered
**Personal planning session**	Each participant met with an activity counselor at enrollment to discuss topics such as readiness to change, barriers, and goal setting.	Not offered	Not offered	Not offered
**Ongoing motivational telephone support**	Activity counselor called participants monthly (more often initially) to discuss progress, barriers, changes in health, and upcoming program workshops.	Not offered	Not offered	Not offered
**New program features**	Not applicable	A program exercise class and walking club were offered weekly. Blood pressure was measured before class, and a tracking log with recommendations such as when exercise may be contraindicated was provided.	Approximately monthly, bilingual physicians provided lectures and question-and-answer sessions related to exercise and various medical conditions.	Not offered

a A new battery of functional fitness tests for older adults was available (35), replacing those in CHAMPS II.

**Table 3:** Characteristics of Participants (N = 321) in the Three Organizations Participating in the Community Healthy Activities Model Program for Seniors (CHAMPS) III^a^

**Characteristic**	**Network for Elders (n = 62) No. (%)**	**On Lok's 30th Street Senior Center (n = 99) No. (%)**	**Sequoia Hospital Health & Wellness Services (n = 160) No. (%)**

**Sex**			

Female	58 (97)	75 (76)	136 (87)
Male	2 (3)	24 (24)	20 (13)

**Education**			

≤6 y	NA^b^	29 (39)	5 (3)
>6 y and less than high school	NA	14 (19)	11 (7)
Completed high school	NA	15 (20)	32 (21)
Some college	NA	9 (12)	57 (38)
College degree or higher	NA	8 (11)	46 (30)

**Age, y**			

<60	3 (5)	4 (5)	1 (1)
60-74	41 (71)	38 (44)	74 (48)
75-84	12 (21)	37 (43)	68 (44)
≥85	2 (3)	8 (9)	12 (8)

**Race or ethnicity**			

Asian	1 (2)	3 (3)	8 (9)
Filipino	0 (0)	4 (4)	0 (0)
African American	53 (90)	0 (0)	8 (9)
Hispanic or Latino	0 (0)	83 (84)	6 (7)
White	5 (8)	8 (8)	63 (67)
Other	0 (0)	1 (1)	8 (9)

a Not all participants answered all questions; percentages may not add to 100 because of rounding.

b NA indicates not applicable; education was not assessed at Network For Elders.

**Table 4:** Changes in Estimated Caloric Expenditure per Week and Hours Spent per Week in Physical Activity, by Organizations in the Community Healthy Activities Model Program for Seniors (CHAMPS) III

**Organization and Cohort**	**N**	**Kcal/week**				**Hours/week**			

		**Baseline Mean (SD)**	**6-Month Follow-up Mean (SD)**	**6-Month Change Mean (SD)**	***P***	**Baseline Mean (SD)**	**6-Month Follow-up Mean (SD)**	**6-Month Change Mean (SD)**	***P***

**Network for Elders**									

Cohort 1	24	2974 (2706)	3808 (3524)	+834 (2363)	.10	11.9 (9.4)	14.7 (11.3)	+2.8 (9.6)	.16
Cohort 2	29	2681 (2160)	2870 (1594)	+189 (1483)	.50	11.5 (9.4)	12.5 (7.0)	+1.0 (6.4)	.39
Total	53	2814 (2403)	3295 (2661)	+481 (1939)	.08	11.7 (9.3)	13.5 (9.2)	+1.9 (8.0)	.10

**On Lok's 30th Street Senior Center**									

Cohort 1	40	2188 (1470)	2059 (1318)	−130 (1239)	.51	10.8 (6.8)	10.2 (6.4)	−0.5 (5.9)	.57
Cohort 2	31	1794 (1305)	1391 (1414)	−403 (1501)	.15	9.2 (7.0)	6.6 (6.5)	−2.6 (8.1)	.08
Total	71	2016 (1405)	1767 (1391)	−249 (1356)	.13	10.1 (6.9)	8.6 (6.6)	−1.4 (7.0)	.09

**Sequoia Hospital Health & Wellness**									

On-site program	6	2544 (1782)	2783 (1673)	+240 (1963)	.77	12.7 (7.1)	15.0 (10.3)	+2.3 (10.6)	.63
Center I, cohort 1	24	3088 (1613)	3484 (1922)	+396 (1796)	.29	14.8 (6.1)	17.1 (8.8)	+2.3 (8.7)	.21
Center I, cohort 2	10	2646 (1579)	4768 (2573)	+2121 (2082)	.01	13.0 (6.5)	22.5 (10.4)	+9.5 (9.3)	.01
Center II, cohort 1	17	2405 (752)	3335 (1984)	+930 (1762)	.04	12.5 (3.7)	16.4 (10.1)	+3.9 (8.5)	.07
Center II, cohort 2	10	4229 (1701)	3547 (1669)	−682 (1392)	.16	23.0 (9.3)	18.5 (7.3)	−4.5 (6.4)	.05
Center III	7	1332 (1211)	1681 (842)	+349 (1082)	.43	6.8 (5.5)	8.6 (4.2)	+1.9 (5.8)	.42
Center IV	9	2910 (2789)	2102 (1689)	−809 (3320)	.49	16.1 (13.5)	12.6 (7.7)	−3.6 (16.9)	.54
Total	83	2826 (1729)	3263 (2004)	+437 (2078)	.06	14.4 (8.1)	16.4 (9.2)	2.0 (10.1)	.08

**Among all organizations**	207	2545 (1861)	2758 (2135)	+213 (1845)	.10	12.2 (8.2)	13.0 (9.0)	+0.8 (8.7)	.20

